# Important Decision in Boston Court

**Published:** 1902-05

**Authors:** 


IMPORTANT DECISION IN BOSTON COURT.—Infringement: Copying style of package. Mess. M. J. Breitenbach Company, proprietors of Gude's Pepto-Mangan, brought suit in February last against Henry Thayer & Co., of Boston, for trespass on their rights, asking that they be enjoined from using the style of package in which Pepto-Mangan, Gude, is put up, the same being original with them and protected by law. The court very promptly in March gave a decision in favor of Breitenbach Co. We copy a portion of decree: Henry Thayer & Co. are "enjoined from making or using in any way, the terra cotta colored wrapper with white letters thereon, and the package in connection therewith, heretofore used by the defendant, for its preparation of iron and manganese, or any other wrapper or package therewith which imitates the wrapper used by the complainant, the M. J. Breitenbach Company for its Gude's Pepto-Mangan, and from selling or offering for sale any preparation of iron and manganese in any package or wrapper of a terra cotta color with white letters of the same style, shape and general arrangement as those of the aforesaid wrapper used by the plaintiff, the M. J. Breitenbach Company, and from using the words "Peptonate-Manganese" on or in connection with such terra cotta colored wrappers with white letters of the same style, shape and general arrangement, as those of plaintiff.

Further, the defendant is directed to forthwith deliver to the plaintiff or its attorneys, to be destroyed, all the terra cotta colored wrappers and packages aforesaid like the said exhibit "B," which the defendant now has on hand or in stock or under its control in any way. And it is further ordered, adjudged and decreed, that the defendant Henry Thayer & Company account to the plaintiff, the M. J. Breitenbach Company for all profits which the said defendant has made from sales of said preparation in its said wrapper and package, and for all profits which the plaintiff would have made in the sales of its Gude's Pepto-Mangan but for the use made by the defendant of its said wrapper and package. and also for the damages to the reputation and standing of the plaintiff's said preparation, known as Gude's Pepto-Mangan, by reason of the said use by the defendants of its said packages and wrappers, and of its preparation therein contained, and for the damages otherwise sustained by the plaintiff by reason of the matters charged in the complaint.

And it is ordered, adjudged and decreed that the plaintiff recover of the defendant the costs of this action.

